# Cutting improves the productivity of lucerne-rich stands used in the revegetation of degraded arable land in a semi-arid environment

**DOI:** 10.1038/srep12130

**Published:** 2015-07-13

**Authors:** Zi-Qiang Yuan, Kai-Liang Yu, Bin-Xian Wang, Wang-Yun Zhang, Xu-Long Zhang, Kadambot H. M. Siddique, Katia Stefanova, Neil C. Turner, Feng-Min Li

**Affiliations:** 1 State Key Laboratory of Grassland Agro-Ecosystem, Institute of Arid AgroEcology, School of Life Sciences, Lanzhou University, Lanzhou, Gansu 730000, China; 2 Department of Environmental Sciences, University of Virginia, Charlottesville, Virginia 22904, USA; 3 The UWA Institute of Agriculture, The University of Western Australia, 35 Stirling Highway, Crawley, WA 6009, Australia; 4 Centre for Plant Genetics and Breeding, School of Plant Biology, The University of Western Australia, M080, 35 Stirling Highway, Crawley, WA 6009, Australia

## Abstract

Understanding the relationships between vegetative and environmental variables is important for revegetation and ecosystem management on the Loess Plateau, China. Lucerne (*Medicago sativa* L.) has been widely used in the region to improve revegetation, soil and water conservation, and to enhance livestock production. However, there is little information on how environmental factors influence long-term succession in lucerne-rich vegetation. Our objective was to identify the main environmental variables controlling the succession process in lucerne-rich vegetation such that native species are not suppressed after sowing on the Loess Plateau. Vegetation and soil surveys were performed in 31 lucerne fields (three lucerne fields without any management from 2003–2013 and 28 fields containing 11-year-old lucerne with one cutting each year). Time after planting was the most important factor affecting plant species succession. Cutting significantly affected revegetation characteristics, such as aboveground biomass, plant density and diversity. Soil moisture content, soil organic carbon, soil available phosphorus and slope aspect were key environmental factors affecting plant species composition and aboveground biomass, density and diversity. Long-term cutting can cause self-thinning in lucerne, maintain the stability of lucerne production and slow its degradation. For effective management of lucerne fields, phosphate fertilizer should be applied and cutting performed.

The Loess Plateau is one of the most seriously-eroded regions in the world, with a poor ecological environment and frequent natural disasters^1^,^2^. The amount of annual soil erosion in the region is estimated at more than 2200 million tonnes per year^3^. Recently, environmental degradation has accelerated due to the increase in population and conversion of natural landscapes to arable land^4^. This raises the issue of revegetation of degraded ecosystems in the region, with natural revegetation being one method to improve ecological conditions^5^,^6^. However, natural revegetation is particularly challenging in semi-arid environments because a long period is usually required to establish stable vegetation cover^7^,^8^; a situation not supported by local officials and farmers on the semi-arid Loess Plateau as it is of little benefit to them^1^. Since 1999, the Chinese government has been implementing a program called ‘Grain for Green’ in western China by converting arable land to other land uses, with the objective of improving vegetation cover and reducing soil erosion^2^.

Lucerne (*Medicago sativa* L.) is a deep-rooted perennial legume that has become an important agricultural forage species worldwide, providing the most feed protein per unit area among forage and grain legumes^9^. It has been planted on the Loess Plateau for more than 2000 years^10^. Recently, lucerne has been widely used for to revegetate degraded land on the Loess Plateau due to its high quality and yield, ability to protect the soil from wind and/or water erosion and to fix atmospheric nitrogen, and adaptability to various climatic and soil conditions^4^,^11^. Planting of lucerne has significantly increased on the Loess Plateau since 1999^10^. Data indicate that about 2.8 million ha of lucerne has been sown in this area, but is far below the actual need for regeneration of vegetation (which for convenience we will call revegetation) and to control soil erosion^12^.

The planting of lucerne on abandoned arable land has resulted in plant communities developing quickly and forming a diverse community of plants in which lucerne is the dominant species that coexists with native later-successional plant species^13^. Some studies have suggested that planting of lucerne can rapidly increase vegetation cover and maintain a high community biomass in the long term (i.e. more than 20 years) on the Loess Plateau^14^,^15^. Biomass in lucerne plant communities has reached 10 t ha^−1^ in this region^14^. However, some studies have shown that lucerne plant communities reach maximum biomass in the first 10 years, after which it gradually declines^14^,^16^. Vegetation cover and biomass are important for controlling soil erosion and for maintaining the soil productive capacity^17^,^18^. In addition, biodiversity is an important index of community characteristics. High species diversity tends to have high community productivity and stability^19^,^20^,^21^,^22^. Therefore, it is necessary to evaluate changes in community characteristics (i.e., aboveground biomass, density of individual plants and diversity index) and factors controlling these characteristics in lucerne revegetation on the Loess Plateau.

Despite recognition of the importance of planting lucerne in this region^14^,^15^, a detailed analysis of important environmental factors controlling revegetation following the planting of lucerne has not occurred, and precludes our ability to understand and evaluate which factors limit the success and sustainability of lucerne planting and which factors control soil erosion. Some studies have shown that soil organic carbon (C) and total nitrogen (N) can change following the sowing of lucerne^23^,^24^, and that this change can feedback on plant community succession^11^,^25^. Could soil organic C and total N be important factors limiting revegetation and soil erosion control following lucerne planting? Moreover, phosphorus (P) and water in soil are considered the primary limiting factors for plant production on the Loess Plateau^4^,^26^. Some studies suggest that lucerne can consume considerable available P in the soil and deep soil water, thus reducing soil available P and water^4^,^27^. However, it is still not clear which factors—such as soil water, soil organic C, N and P—are more important in a lucerne plant community. In addition, topographic characteristics (e.g. elevation, slope and aspect) affect soil properties, moisture and temperature, and ultimately the distribution of plant species^28^. It has been shown that vegetation, soil nutrient content and topography on the Loess Plateau are highly correlated^29^. Because of its value as an animal feed, particularly if preserved for winter feed, stands of lucerne are frequently cut by farmers in autumn. Cutting is an important factor that can affect the lucerne plant community. Studies have shown that different cutting frequency and strength have different effects on the mixture of species in the community, dry matter distribution and lucerne yield^30^,^31^. Given the above, understanding the long-term influence of soil variables, topographic variables and cutting on the lucerne plant community will be helpful in developing revegetation and management strategies on the Loess Plateau and other regions, such as North America and many European countries, where this species is extensively planted^9^.

The objectives of this study were to analyze the effects of environmental factors (i.e. annual precipitation, topography and soil properties), time after planting and cutting on species composition, aboveground biomass, and the density and diversity of lucerne plant communities, and to identify the key factors controlling these community characteristics on the Loess Plateau. The ultimate goal was to provide a scientific basis for improved management and revegetation decisions for lucerne-rich plant communities, and to promote the sustainability of lucerne planting for revegetation in the semi-arid loess area.

## Results

### Relationships between species abundance and environmental factors

The redundancy analysis (RDA) results showed a strong correlation between species data and environmental factors, with species–environment correlations on both the first and second axes (Table 1). Environmental variables significantly explained total variance and the variation along the first ordination axis (Table 1). Marginal effects on the eigenvalues of explained variance in the RDA indicated that the best explanatory variables for species composition were time after planting, cutting, precipitation, soil moisture, available P, soil organic C, slope, slope position and soil total N (Table 2). According to the Monte Carlo test, the conditional effects indicated that time after planting, soil moisture content, soil organic C, available P, cutting, and slope aspect were significant in the total sum of eigenvalues during the forward selection, and accounted for 91.9% of the total variance explained by environmental factors (Table 2).

**Figure 1:**
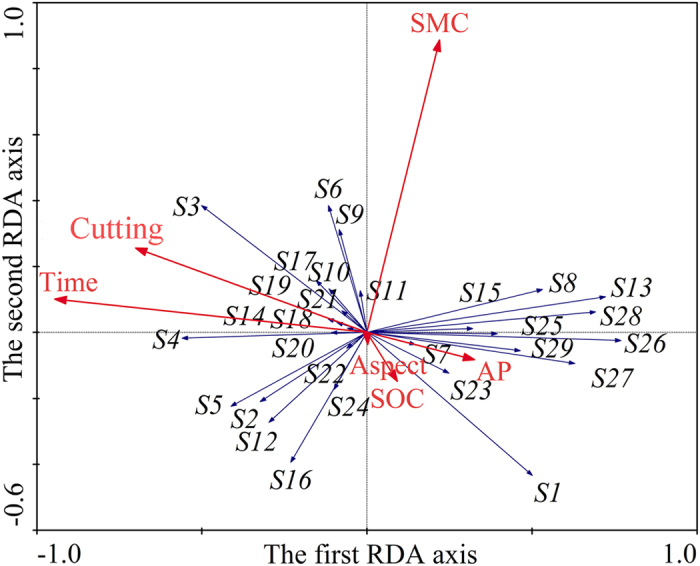
Ordination diagram showing the results of redundancy analysis of species abundance and key experimental variables. Time, time after planting; SMC, soil moisture content; Aspect, slope aspect; SOC, soil organic C; AP, soil available P. Arrows (*S1*–*S28*) represent plant species (see supplementary Appendix 1).

Time after planting was the dominant environmental variable correlated with the first RDA axis (Fig. 1). Soil moisture content was the dominant environmental variable correlated with the second RDA axis. Annual species such as *Convolvulus arvensis*, *Setaria viridis*, *Corispermum declinatum* and *Chenopodium glaucum* were the pioneer species in the lucerne plant communities, and were negatively correlated with time after planting; they mainly appeared in communities in the first or second year after planting (Fig. 1). Perennial species such as *Artemisia frigida*, *Heteropappus altaicus* and *Stipa capillata* were positively correlated with time after planting, as they mainly appeared in later sampling years (Fig. 1).

### Relationships between vegetation variables and environmental factors

**Figure 2:**
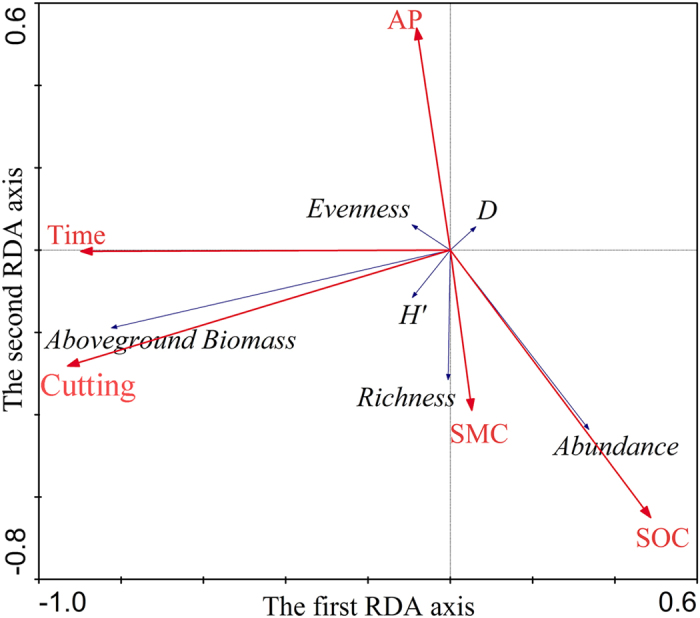
Ordination diagram showing the results of redundancy analysis of vegetation variables (aboveground biomass, density and diversity) and key experimental factors. For Time, AP, SMC and SOC see Fig. 1.

The eigenvalues for all canonical axes and for the first axis of the RDA were significant (Table 3). Experimental factors explained >49% of the variation in vegetation data (Table 3). Cutting, soil organic C, time after planting, soil available P and soil moisture content significantly explained the variation in vegetation variables (Table 4). The key environmental factors for vegetation variables were similar to those affecting species distribution, but their contributions differed (Tables 2 and 4). The ordination diagram of key environmental factors and vegetation variables showed that time after planting and cutting were the dominant environmental variables correlated with the first RDA axis; and soil moisture content, soil available P and soil organic C were the dominant environmental variables correlated with the second axis (Fig. 2).

**Figure 3:**
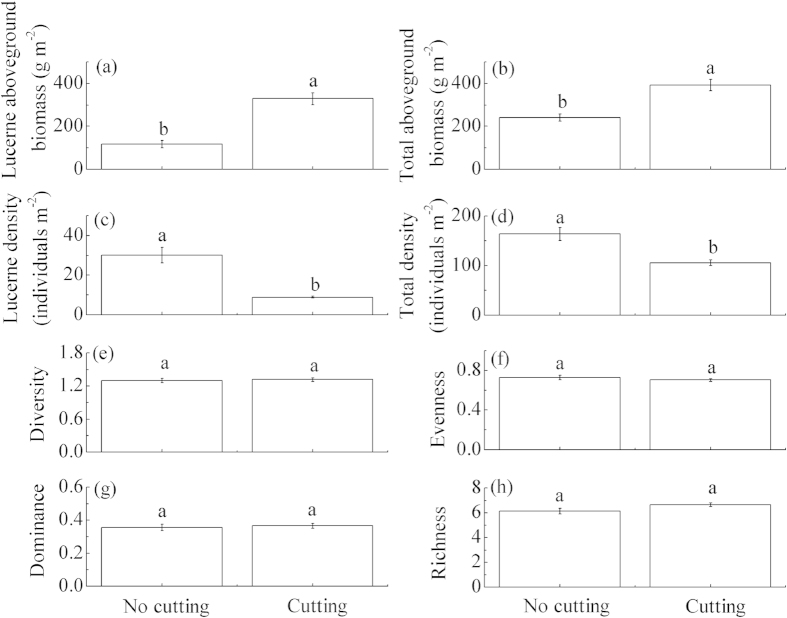
Aboveground biomass (g m^−2^), density, diversity, evenness, dominance and richness in non-cut (means ± SD, *n* = 30) and cut 11-year-old lucerne-rich fields (means ± SD, *n* = 112) in 2013 on the Loess Plateau. The different letters indicate significant differences at *P* < 0.05 in non-cut and cut lucerne-rich fields.

In 2013, the aboveground biomass of lucerne and the whole plant community were significantly higher in the cut than the non-cut fields, but the densities of lucerne and the whole plant community were significantly lower (Fig. 3a–d). There were no significant differences in the Shannon–Wiener index, evenness index, dominance index and species richness between the cut and non-cut fields (Fig. 3e–h).

## Discussion

Arable land converted into pasture can increase vegetation cover, biomass and improve the ability to protect the soil from wind and/or water erosion^8^,^32^,^33^,^34^. Pastures can be used to develop animal husbandry, thereby increasing the income of local farmers, reducing their excessive land reclamation, and indirectly increasing vegetation cover and improving the control of soil erosion^35^. Since 1999, large areas of arable land on the Loess Plateau have been converted to pasture, and lucerne has been used to revegetate the land^2^,^36^. However, effective assessment of the relationships between the revegetation with lucerne and environmental factors is not available. In this study, we evaluated the relationships between lucerne-rich plant communities and environmental factors, and detected the key factors which control species composition, aboveground biomass, density and diversity of lucerne-rich plant communities.

Some studies have shown that time is the most important factor for species succession in the natural establishment of vegetation in the semi-arid region^37^,^38^. Natural revegetation requires about 40 years to establish stable vegetation cover in regions with serious soil erosion on the semi-arid Loess Plateau^39^. In this study, time after planting was the most important experimental factor for species succession, which significantly explained the vegetation variables. Lucerne is a deep-rooted perennial legume that can live for several decades on the semi-arid Loess Plateau^11^. Its productivity reaches a maximum 6–8 years after seeding, and then gradually degenerates^40^. As the lucerne community develops, some species disappear and other native perennial species with better adaptability gradually establish—so time of planting is important for changes in plant species.

Cutting is a strong selective force in pastures due to changes in competitive relationships between plant species from altered light availability within a canopy, damage of foliage and flowers, and the prevention of litter accumulation^41^. Results from this study suggest that cutting in autumn was the most important environmental factor influencing lucerne vegetation characteristics (aboveground biomass, plant density and diversity) which is consistent with many studies^41^,^42^,^43^. Moderate cutting can influence soil resource use by lucerne roots by inhibiting longitudinal growth of taproots and promoting lateral growth^44^,^45^, and can also increase the regeneration ability and production of lucerne aboveground biomass^31^,^45^. Thus, cutting can significantly affect lucerne vegetation characteristics. Moderate grazing or mowing is considered an important intervention method to maintain pasture diversity and productivity. In this case, the aboveground biomass of lucerne was significantly higher (280%) in the cut fields compared with non-cut fields in 11-year-old lucerne communities. This result indicates that moderate cutting in a lucerne-rich pasture can maintain the stability of lucerne production and slow its degradation, and is important for revegetation. Thus we suggest that moderate cutting serves as a management measure for revegetation using lucerne on the semi-arid Loess Plateau.

Our results indicate that soil moisture content is also a key factor affecting species succession and vegetation characteristics of lucerne-rich communities, which is consistent with some studies^26^,^46^. Precipitation in the semi-arid region of the Loess Plateau is limited with an average of 300 mm, and large year-to-year fluctuations^35^. The actual evapotranspiration of lucerne stands is far greater than precipitation during the growing season^10^ and growth of perennial lucerne can lead to desiccation of deep soil layers in the hilly region of the Loess Plateau^15^. Soil desiccation is considered the main reason for lucerne degradation^40^. Li and Huang (2008) found that reduced soil water availability resulted in the pasture yield responding more to variation in annual precipitation on the Loess Plateau^10^. We did not find a significant effect of precipitation on lucerne vegetation (Tables 2 and 4) which may be because the period of high rainfall (June–September) does not coincide with the lucerne-growing season (March–November) on the Loess Plateau.

The conversion of arable land to pasture can significantly improve soil quality, and this improvement can feedback on plant community succession^47^. The present study showed that soil properties (soil organic C and available P) had significant and greater effects than topography (slope, aspect and slope position) on species succession (Table 2) and vegetation characteristics (Table 4). These results further support the observation that soil organic C and nutrients largely determine plant establishment, growth and distribution^48^,^49^. Soil organic C is a critical component of the soil base and plays an important role in maintaining ecosystem productivity^50^. Li *et al.* (2006) found that the planting of lucerne enhanced soil organic C in fields without fertilization and cutting^13^. However, some studies have found that soil organic C initially decreased and then increased in non-fertilized and non-cut lucerne fields^51^. These results suggest that the dynamics of soil organic C in lucerne fields are complex and require more detailed study. P is the main limiting factor in determining both biomass production and botanical change on the Loess Plateau^26^. In this study, soil available P was about 5 mg kg^−1^ and the ratio of soil organic C to available P was >1000 in the 11-year-old lucerne-rich communities. These results suggest that the soils were facing a serious deficiency of available P, and that soil microorganisms were likely competing with plant species for soil available P^52^. Thus, to maintain lucerne production in the long term, an appropriate amount of P fertilizer should be applied.

Topography can partly affect the accumulation and export of soil nutrients, thereby indirectly impacting plant distribution^46^. Our results indicate that slope aspect, but not slope position or slope, significantly affected plant species, which is consistent with some previous results^26^,^29^,^53^. Research has shown that slope aspect and position affect soil chemical properties, litter quality and nutrient cycling^54^. Generally, the soil nutrient pool is greater in north-facing than south-facing slopes due to less organic matter decomposition and greater accumulation of organic C and total N on cold, north-facing slopes^55^. Lucerne, a legume with high water consumption, is sensitive to changes in soil moisture and available P^51^,^52^. Thus, topographic variables (such as slope aspect) and soil properties can interact with each other to affect plant species distribution in lucerne-rich communities^56^.

We conclude that to maintain the long-term productivity of lucerne-rich plant communities, some management measures such as applying P fertilizer and moderate cutting is required. It should be noted that 11 years after lucerne was sown, the fields in this study still maintained high biomass levels. We therefore suggest that lucerne planting should be continued for vegetation restoration and to control soil erosion on the Loess Plateau.

## Methods

### Study area

This study was conducted at the Ecological Research Station of Lanzhou University in the northern mountainous region of Yuzhong County, Gansu, China (104°24′E, 36°02′N; 2400 m above sea level). Mean annual temperature is 6.5 °C, ranging from −8.0 °C in January to 19 °C in July. The area has a semi-arid steppe climate with mean annual precipitation of 301 mm, with approximately 60% occurring from June–September. Average annual open-pan evaporation is about 1300 mm. The soil is Heima (Calcic Kastanozems, FAO Taxonomy) with a field water holding capacity of 23% by weight and permanent wilting point of 4.5% by weight^57^.

### Experimental design

**Figure 4:**
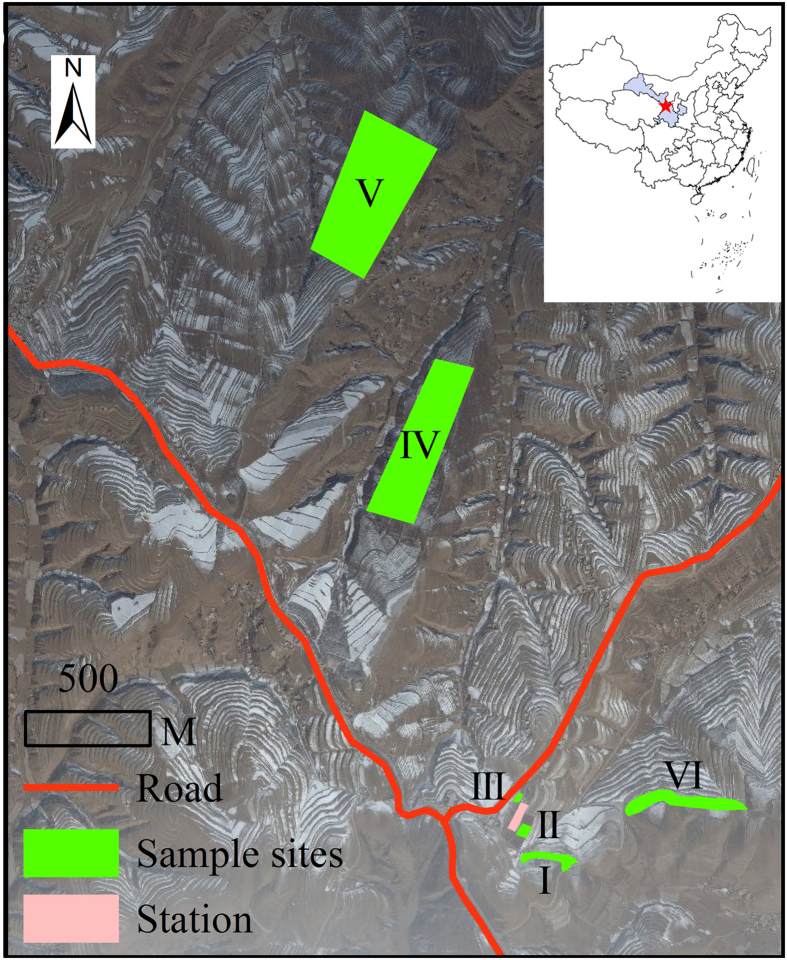
The sampling sites (I–VI) of lucerne (*Medicago sativa*) fields on the Loess Plateau. Three continuously-cultivated lucerne-rich fields without cutting were located at sites I–III; 28 lucerne fields mown once per year were located at sites IV–VI. The software ArcMap (version 10.0, ESRI) was used to create the map.

Three lucerne-rich fields (more than 35 m × 40 m per field), one faced north-east with a slope of 10–14°, while the other two faced south-east with slopes of 12–16° and 4–8°, were established in April 2003 at a seed density of 22.5 kg ha^−1^ (Fig. 4). After establishment, the fields had no grazing, no tillage, no fertilization, no cutting, mowing or harvesting, or other management.

At the beginning of August 2013, 28 lucerne-rich hillslope fields were selected from the same valley near the Ecological Research Station of Lanzhou University (Fig. 4). Lucerne had been planted in these fields in April 2003 at the same seed density (according to the village head who conducted the lucerne planting) as the three lucerne-rich fields. From 2004 onwards, the 28 lucerne fields were mown to the soil surface at the beginning of October each year, and the aboveground biomass removed, with no additional management until harvest in August 2013.

### Vegetation and soil sampling

In the three non-cut lucerne-rich fields, ten quadrats (1 m × 1 m) were randomly placed in each field at the beginning of August each year (from 2003–2013). To avoid edge effects, all quadrats were at least 3 m from field boundaries. In each quadrat, the number of individuals of each species were counted to determine the Shannon–Wiener diversity index, Pielou’s evenness index and Simpson’s predominance index^45^,^58^. Aboveground biomass was assessed as the weight of all plant species in a quadrat by cutting all plants at the soil surface and oven-drying to constant weight at 80 °C for 48 h. Three replicated soil cores (8 cm diameter × 20 cm depth) were randomly collected in each field in August each year from 2003–2013. At the same time, three additional soil samples per field were collected for measuring soil moisture to a depth of 2.0 m in increments of 0.2 m. Soil moisture content was determined gravimetrically by drying samples at 105 °C for 10 h. The slope position of each field was recorded, while the slope and slope aspect were measured using a compass.

In the 28 cut lucerne-rich fields, four sampling quadrats (1 m × 1 m) were randomly placed in each field at the beginning of August 2013. In each quadrat, the numbers of individuals of each species were counted to determine the Shannon–Wiener diversity index, Pielou’s evenness index and Simpson’s predominance index. Aboveground biomass was assessed as per the non-cut fields. Three soil samples at a depth of 0.2 m were randomly collected and bulked to obtain a composite sample and soil moisture determined to a depth of 2.0 m as described above. The slope, slope aspect and position of each quadrat were also measured.

Each soil sample was air-dried to estimate soil parameters. Available P was extracted by the Olsen method^59^. Total P was determined by molybdate colorimetric method after perchloric acid digestion and ascorbic acid reduction^60^. Soil organic C was determined by the Walkley–Black method^61^. A KJ (Kjeldahl) Auto Analyzer (TECATOR Product, Sweden) was used to measure total soil N after digestion with salicylic acid-H_2_SO_4_.

### Statistical analyses

Multivariate analyses were performed using CANOCO 4.5^62^ to explore the relationships between (i) species abundance and environmental factors, and (ii) vegetation variables (aboveground biomass, species richness, Shannon–Wiener diversity index, Pielou’s evenness index, Simpson’s predominance index and total abundance) and environmental factors. Environmental variables were the same in these two multivariate analyses: i.e. soil total N, soil total P, soil available P, soil organic C, time after planting (year), yearly precipitation, soil moisture content at 0–0.2 m, cutting, slope aspect, slope position and slope. The soil variables (total N, total P, available P, organic C and moisture content) of the three soil cores within a field were fairly homogeneous in the three non-cut lucerne fields so they were averaged and then used for correspondence with species data in each field. In the 28 cut lucerne fields, the data in each quadrat per field were used. The multivariate analysis used eight classes of aspect: 1 (337.6°–22.5°), 2 (22.6°–67.5°), 3 (292.6°–337.5°), 4 (67.6°–112.5°), 5 (247.6°–292.5°), 6 (112.6°–157.5°), 7 (202.6°–247.5°) and 8 (157.6°–202.5°)^53^, and four classes of slope position: 1 (top), 2 (upper), 3 (middle) and 4 (bottom).

In this study, the results of de-trended correspondence analysis showed that the longest gradient length was 2.622 for the data of species abundance and 0.391 for the data of vegetation variables. A linear model with RDA was most useful when the gradient length was <3, while a unimodal model with canonical correspondence analysis was suitable when it was >4; for intermediate lengths, both models can be useful^63^. Thus, we performed an RDA to explore the relationships between environmental factors and vegetation^62^. A Monte Carlo permutation test based on 499 random permutations was conducted to test the significance of the eigenvalues of all canonical axes. To identify the contribution of environmental factors to explain species data, the eigenvalues and statistical significance of each variable in the analyses of environmental variables alone (marginal effects) and forward selection of environmental variables (conditional effects) were assessed by Monte Carlo permutation tests (499 permutations)^62^.

The data were unbalanced, with 28 fields with cutting with four replicates compared to three fields without cutting with 10 replicates. A linear mixed model (LMM) accommodates unbalanced data and therefore was used to assess the effect of cutting the 11-year-old lucerne-rich fields on the following vegetation variables: aboveground biomass, density, diversity, evenness and dominance. The only response variable analyzed using a generalized linear mixed model (GLMM) was richness. A Poisson distribution was assumed for the latter and respectively a log function was used as a link function in the analysis. For all models, the experimental design was accounted for, namely the fields and interaction of fields by replicate were fitted as random. The replicate was the sample quadrat randomly selected within each field. All of the abovementioned analyses were conducted using GenStat 17^th^ Edition (VSN Int. 2014).

## Additional Information

**How to cite this article**: Yuan, Z.-Q. *et al.* Cutting improves the productivity of lucerne-rich stands used in the revegetation of degraded arable land in a semi-arid environment. *Sci. Rep.* **5**, 12130; doi: 10.1038/srep12130 (2015).

## Supplementary Material

Supplementary Information

## Figures and Tables

**Table 1:** Results of redundancy analysis on species abundance and environmental factors for *Medicago sativa* fields on the Loess Plateau.

**Axes**	**1**	**2**	**3**	**4**	**Total variance**
Eigenvalues	0.214	0.06	0.035	0.017	1.000
Species–environment correlations	0.888	0.669	0.593	0.562	
Cumulative percentage variance of species data	21.4	27.4	30.8	32.6	
Cumulative percentage variance of species–environment relations	60.2	77.0	86.7	91.5	
Sum of all eigenvalues					1.000
Sum of all canonical eigenvalues					0.356
Test of significance of first canonical axis	Trace	F-ratio	P-value		
	0.214	33.824	0.002		
Test of significance of all canonical axes	Trace	F-ratio	P-value		
	0.356	6.847	0.002		

**Table 2:** The percentage variation (V) in species data explained by environmental variables in a redundancy analysis (RDA) estimated using two different methods: V_1_ marginal effects (one variable at a time) and V_2_ conditional effects (forward selection of variables).

**Variable**	**λ_1_**	**V_1_**	**λ_2_**	**V_2_**
Time after planting	0.194**	19.4	0.194**	54.3
Soil moisture content	0.057**	5.7	0.05**	14.0
Soil organic C	0.024**	2.4	0.033**	9.24
Soil available P	0.04**	4	0.025**	7.00
Cutting	0.115**	11.5	0.015**	4.20
Slope aspect	0.009	0.9	0.011*	3.08
Soil total P	0.006	0.6	0.009	2.52
Precipitation	0.09**	9	0.008	2.24
Slope	0.022**	2.2	0.007	1.96
Slope position	0.019*	1.9	0.004	1.12
Soil total N	0.017*	1.7	0.001	0.28

λ_1_ = eigenvalue of first axis in a RDA with one environmental variable. V_1_ = [λ_1_/sum of all unconstrained eigenvalues in a RDA, total inertia] × 100 (percentage variation in species data explained by environmental variable alone, marginal effects). λ_2_ = eigenvalue of first axis in a RDA with forward selection of the environmental variables. V_2_ = [λ_2_/sum of all canonical eigenvalues in a RDA] × 100 (percentage variation in species data explained by the variable in a forward selection where the variation explained by more important variables is removed, conditional effects).

*, ** significant at P < 0.05 and P < 0.01 in Monte Carlo permutation tests, respectively.

**Table 3:** Results of redundancy analysis of vegetation variables (aboveground biomass, density and diversity) and environmental factors on *Medicago sativa* fields on the Loess Plateau.

**Axes**	**1**	**2**	**3**	**4**	**Total variance**
Eigenvalues	0.360	0.121	0.012	0.002	1.000
Species–environment correlations	0.824	0.572	0.373	0.313	
Cumulative percentage variance of species data	36.0	48.1	49.2	49.4	
Cumulative percentage variance of species–environment relations	72.8	97.3	99.6	100.0	
Sum of all eigenvalues					1.000
Sum of all canonical eigenvalues					0.494
Test of significance of first canonical axis	Trace	F-ratio	P-value		
	0.360	69.733	0.002		
Test of significance of all canonical axes	Trace	F-ratio	P-value		
	0.494	12.114	0.002		

**Table 4:** Percentage variation (V) in community traits explained by environmental variables in a redundancy analysis (RDA) estimated using two different methods: V_1_ marginal effects (one variable at a time) and V_2_ conditional effects (forward selection of variables).

**Variable**	**λ_1_**	**V_1_**	**λ_2_**	**V_2_**
Cutting	0.309**	30.9	0.309**	62.6
Soil organic C	0.129**	12.9	0.081**	16.4
Time after planting	0.281**	28.1	0.038**	7.70
Soil available P	0.035*	3.5	0.026**	5.26
Soil moisture content	0.019	1.9	0.012*	2.43
Soil total P	0.003	0.3	0.009	1.82
Precipitation	0.151	15.1	0.008	1.62
Slope	0.037**	3.7	0.007	1.42
Slope position	0.011	1.1	0.002	0.40
Slope aspect	0.017	1.7	0.001	0.20
Soil total N	0.063*	6.3	0.001	0.20

λ_1_ = eigenvalue of first axis in a RDA with one environmental variable. V_1_ = [λ_1_/sum of all unconstrained eigenvalues in a RDA, total inertia] × 100 (percentage variation in species data explained by environmental variable alone, marginal effects). λ_2_ = eigenvalue of first axis in a RDA with forward selection of environmental variables. V_2_ = [λ_2_/sum of all canonical eigenvalues in a RDA] × 100 (percentage variation in species data explained by the variable in a forward selection where the variation explained by more important variables is removed, conditional effects).

*, ** significant at P < 0.05 and P < 0.01 in Monte Carlo permutation tests, respectively.
