# Supplementary material for: Cutting improves the productivity of lucerne-rich stands used in the revegetation of degraded arable land in a semi-arid environment
Source: Sci Rep. 2015 Jul 13;5:12130. doi: 10.1038/srep12130 (PMC4499809; doi:10.1038/srep12130)
Supplement: Supplementary Information [file srep12130-s1.doc]

**Cutting improves the productivity of lucerne-rich stands used in the revegetation of degraded arable land in a semi-arid environment**

Zi-Qiang Yuan, Kai-Liang Yu, Bin-Xian Wang, Wang-Yun Zhang, Kadambot H.M. Siddique, Katia Stefanova, Neil C. Turner & Feng-Min Li

**Appendix 1** Information on taxonomy, life form and functional group of the 28 species that appeared in *Medicago sativa* L. fields. Taxonomy follows Wu et al (1994–2011)^64^.

|  | Species | Family | Life form | Functional group |
| --- | --- | --- | --- | --- |
| *S1* | *M****ed****icago. sativa* L. | Leguminosae | Perennial | Legume |
| *S2* | *Agropyron cristatum* (L.) Gaertn. | Gramineae | Perennial | Grass |
| *S3* | *Artemisia frigida* Willd. | Compositae | Perennial | Forb |
| *S4* | *Heteropappus altaicus* (Willd.) Novop | Compositae | Perennial | Forb |
| *S5* | *Stipa grandis* P. Smirn. | Gramineae | Perennial | Grass |
| *S6* | *Anaphalis lacteal* Maxim. | Asteraceae | Perennial | Forb |
| *S7* | *Potentilla bifurca* L. | Rosaceae | Perennial | Forb |
| *S8* | *Convolvulus arvensis* L. | Convolvulaceae | Annual | Forb |
| *S9* | *Poa annua* L. | Gramineae | Perennial | Grass |
| *S10* | *Leymus secalinus* (Georgi) Tzvel. | Gramineae | Perennial | Grass |
| *S11* | *Taraxacum pingue* Schischk. | Compositae | Perennial | Forb |
| *S12* | *Saussurea pulchella* Fisch. ex DC. | Compositae | Perennial | Forb |
| *S13* | *Setaria viridis* (L.) Beauv. | Gramineae | Annual | Forb |
| *S14* | *Polygonum nepalense* Meisn. | Polygonaceae | Perennial | Forb |
| *S15* | *Elsholtzia ciliata* (Thunb.) Hyland. | Labiatae | Annual | Forb |
| *S16* | Torularia humilis (C.A.Meyer) O.E. Schulz. | Cruciferae | Perennial | Forb |
| *S17* | *Heteropappus altaicus* (Willd.) Novopokr. | Compositae | Perennial | Forb |
| *S18* | *Herba Artimisiae* Sieversianae | Asteraceae | Annual | Forb |
| *S19* | *Achnatherum breviaristatum* Keng et P. C. | Poaceae | Perennial | Grass |
| *S20* | Oxytropis ochrocephala Bunge | Leguminosae | Perennial | Legume |
| *S21* | *Astragalus polycladus* Bur. et Franch. | Leguminosae | Perennial | Legume |
| *S22* | *Potentilla fragarioides* L. | Rosaceae | Perennial | Forb |
| *S23* | *Salsola collina* Pall. | Chenopodiaceae | Annual | Forb |
| *S24* | *Lappula myosotis* V. Wolf | Boraginaceae | Annual | Forb |
| *S25* | *Sonchus oleraceus* L. | Compositae | Annual | Forb |
| *S26* | *Corispermum declinatum*Steph. ex Stev. | Chenopodiaceae | Annual | Forb |
| *S27* | *Chenopodium glaucum* L. | Chenopodiaceae | Annual | Forb |
| *S28* | *Polygonum nepalense* Meisn. | Polygonaceae | Perennial | Forb |

^64^ Wu, Z.Y., Raven, P.H. & Hong, D.Y. (Eds.) Flora of China. Science Press, Missouri Botanical Garden Press, Beijing, St. Louis (1994-2013).
